# Tuneable Magnetic Phase Transitions in Layered CeMn_2_Ge_2-x_Si_x_ Compounds

**DOI:** 10.1038/srep11288

**Published:** 2015-06-19

**Authors:** M. F. Md Din, J. L. Wang, Z. X. Cheng, S. X. Dou, S. J. Kennedy, M. Avdeev, S. J. Campbell

**Affiliations:** 1 Institute for Superconductivity and Electronic Materials, University of Wollongong, Wollongong, NSW 2522, Australia; 2 Bragg Institute, Australian Nuclear Science and Technology Organization, Lucas Heights, NSW 2234, Australia; 3 School of Physical, Environmental and Mathematical Sciences, The University of New South Wales, Canberra, ACT 2600, Australia; 4 Department of Electrical & Electronic Engineering, Faculty of Engineering, National Defence University of Malaysia, Kem Sungai Besi, 57000 Kuala Lumpur, Malaysia

## Abstract

The structural and magnetic properties of seven CeMn_2_Ge_2-x_Si_x_ compounds with x = 0.0–2.0 have been investigated in detail. Substitution of Ge with Si leads to a monotonic decrease of both a and c along with concomitant contraction of the unit cell volume and significant modifications of the magnetic states - a crossover from ferromagnetism at room temperature for Ge-rich compounds to antiferromagnetism for Si-rich compounds. The magnetic phase diagram has been constructed over the full range of CeMn_2_Ge_2-x_Si_x_ compositions and co-existence of ferromagnetism and antiferromagnetism has been observed in CeMn_2_Ge_1.2_Si_0.8_, CeMn_2_Ge_1.0_Si_1.0_ and CeMn_2_Ge_0.8_Si_1.2_ with novel insight provided by high resolution neutron and X-ray synchrotron radiation studies. CeMn_2_Ge_2-x_Si_x_ compounds (x = 0, 0.4 and 0.8) exhibit moderate isothermal magnetic entropy accompanied with a second-order phase transition around room temperature. Analysis of critical behaviour in the vicinity of T_C_^inter^ for CeMn_2_Ge_2_ compound indicates behaviour consistent with three-dimensional Heisenberg model predictions.

Due to the wide and interesting range of structural and magnetic phenomena - including magnetism, superconductivity, mixed valence, Kondo behaviour and heavy fermion - exhibited by layered RT_2_X_2_ rare earth compounds, (R is a rare earth, T is transition metal and X is Si or Ge) this series has attracted significant attention over the years (e.g.^1^,^2^,^3^,^4^). Most of the layered RT_2_X_2_ crystallize in the body centred tetragonal ThCr_2_Si_2_-type structure with space group *I4/mmm* in which the R, T and X atoms occupy the 2*a*, 4*d* and 4*e* sites respectively, with the different atoms stacked along the *c*-axis in the layered sequence R–X–T–X–R^5^,^6^,^7^. An important factor in the continued interest in the RMn_2_X_2_ over the past two decades is that the magnetic states of the Mn-sublattice depend sensitively on the inter-planar and intra-planar Mn–Mn distances^8^,^9^. Furthermore, intensive studies of RMn_2_X_2_ compounds have revealed a large variety of magnetic structures and magnetic phase transitions that occur with changes in chemical composition, temperature and mechanical pressure or magnetic field applied^10^,^11^,^12^. From this point of view, RMn_2_X_2_ compounds provide model systems for study of, for example, the volume dependence of magnetic ordering. They also offer scope for design of critical magnetic parameters — such as the type or order of magnetic phase transitions and ability to shift transition temperatures — by controlling the intra-planar separation distance *d*^*a*^_*Mn-Mn*_ with applied mechanical or chemical pressure via replacement with elements of different atomic sizes^13^,^14^,^15^.

In this study, we report the findings of an investigation of the effects of substituting Si for Ge in CeMn_2_Ge_2-x_Si_x_ (x = 0.0–2.0) on their magnetic properties and structures using magnetic, differential scanning calorimetry (DSC), high resolution X-ray synchrotron radiation and neutron diffraction measurements. In addition to exploring the effects of the Si atoms on the metalloid Ge, it is expected that substitution of Ge (atomic radius 1.37 Å) with the smaller Si (atomic radius 1.32 Å) would modify the magnetic structures of CeMn_2_Ge_2-x_Si_x_. This follows as *d*^*a*^_*Mn-Mn*_ ∼ 2.93 Å in CeMn_2_Ge_2_ while *d*^*a*^_*Mn-Mn*_ ∼ 2.83 Å in CeMn_2_Si_2_^16^ (*d*^*a*^_*Mn-Mn*_ is the Mn–Mn separation distance in the *ab*-plane). These values of *d*^*a*^_*Mn-Mn*_ are respectively greater than *d*_crit1_ ∼ 2.87 Å (of related lattice parameter a_crit1_ = 4.06 Å) and less than *d*_crit2_ ∼ 2.84 Å (related lattice parameter a_crit2_ = 4.02 Å), the first and second critical intralayer Mn–Mn distances which govern the magnetic behaviour in RMn_2_X_2_ compounds^17^,^18^. According to Welter *et al.*^19^, the *d*^*a*^_*Mn-Mn*_ not only affects the intralayer Mn–Mn coupling but also the interlayer exchange interaction and, as summarised below, three general categories can be delineated^20^.

(i) *d*^*a*^_*Mn-Mn*_ *>* *d*_crit1_ = 2.87 Å: The interlayer exchange coupling is ferromagnetic and the intralayer coupling antiferromagnetic; this leads to the canted Fmc-type ferromagnetic structure (see detailed definition of magnetic structures in Figure S1 of the Supplementary Material; the Fmc structure can be described by the Im-m2- magnetic space group (Opechowski-Guccione #44.3.326, basis (b,c,a;0 0 0); active vector (0,0,0)).

(ii) *d*_crit2_ = 2.84 Å *<* *d*^*a*^_*Mn-Mn*_ *<* *d*_crit1_ = 2.87 Å; Both the interlayer and the intralayer coupling are antiferromagnetic; this leads to the AFmc-type magnetic structure. AFmc structure can be described by the Pnnm’ magnetic space group (Opechowski-Guccione #58.4.474, basis (-a, c, b; 0,0,0); active vector (0,0,1)).

(iii) *d*^*a*^_*Mn-Mn*_ *<* *d*_crit2_ = 2.84 Å; No intralayer in-plane spin component and antiferromagnetic interlayer coupling; this leads to the AFil-type magnetic structure which can be described by I_P_4/m’m’m’ magnetic space group (Opechowski-Guccione #139.17.1195; basis (a, b, c; 1/4, 1/4, 1/4); active vector (0,0,1)).

The interest in the properties and behaviour of layered structure materials has been enhanced in recent years by the discovery of a giant magnetocaloric effect (MCE) near room temperature in Gd_5_Si_2_Ge_2_ compound^21^,^22^,^23^. R_5_(Si,Ge)_4_ type compounds have a distinct layered structure in which the covalent Si–Si, Si–Ge and Ge–Ge bonds and interlayer distances play a vital role in determining their magnetic and magnetocaloric effect properties^22^. This in turn has led to an increased focus on understanding the fundamental properties of this type of layered material. As already noted, the CeMn_2_Ge_2-x_Si_x_ system has a sequence of atomic layers stacked along the *c*-axis similar to the R_5_(Si,Ge)_4_ system. Also as noted, the relatively simple body centred tetragonal ThCr_2_Si_2_-type structure offers scope for selection of the magnetic state via the strong dependence of Mn–Mn intraplanar and interplanar exchange interactions on *d*^*a*^_*Mn-Mn*_ the Mn–Mn separation distance in the *ab*-plane. This information is expected to provide enhanced understanding of the correlation between magnetic properties and atomic distances. Our comprehensive investigation of the crystallographic, magnetic properties and critical exponent behaviour of CeMn_2_Ge_2-x_Si_x_ compounds (x = 0.0–2.0) has enabled us to establish the magnetic structures across the Ge-Si concentration range and derive the magnetic phase diagram of CeMn_2_Ge_2-x_Si_x_.

## Results

### Crystal structure and magnetic phase transitions

**Figure 1:**
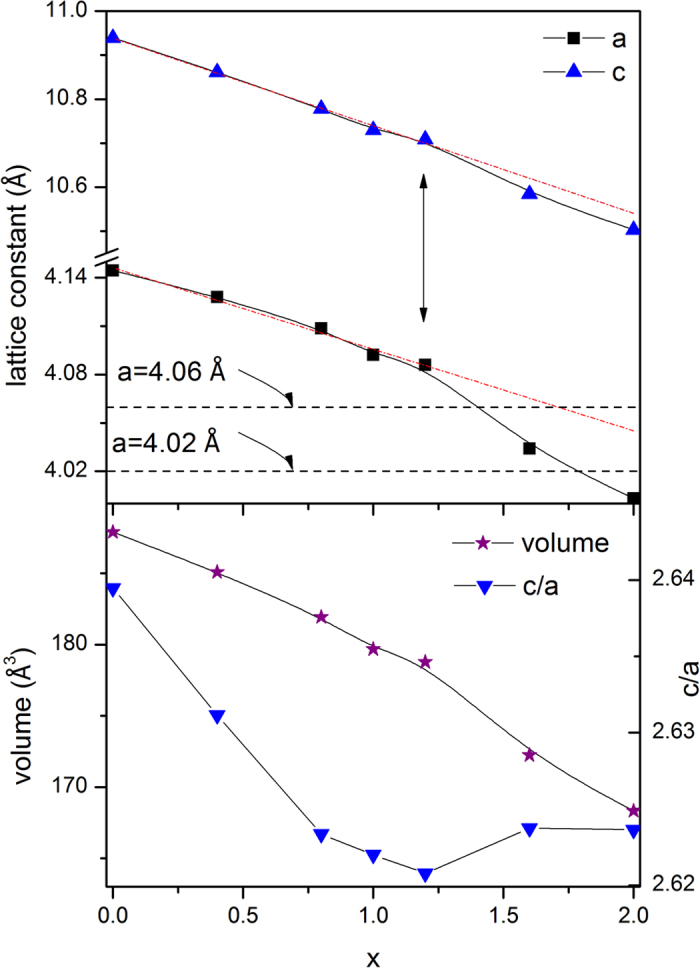
Composition dependence of lattice parameters *a* and *c*, axial ratio *c*/*a* and unit cell volume, V, for CeMn_2_Ge_2-x_Si_x_ at room temperature. The arrows indicate the region where the slope changes with the dotted line denoting behaviour consistent with Vegard’s law. The full lines are guides to the eye with the dashed lines of the upper figure denoting the a_crit1_ ~ 4.06 Å and a_crit2_ ~ 4.02 Å values as explained in the text.

Rietveld refinements (FULLPROF package^24^) of the room temperature X-ray diffraction patterns indicate that all of the CeMn_2_Ge_2-x_Si_x_ samples crystallize in the ThCr_2_Si_2_ structure. The refined results — including lattice parameters *a*, *c*, axial ratio *c*/*a* and unit cell volume V — are shown in Fig. 1. As expected, substitution of Ge by Si leads to a monotonic decrease of both *a* and *c* along with concomitant contraction of the unit cell volume with increasing Si content; good agreement is obtained with published results^16^. However, it is noted that the variations of the lattice parameters and unit cell volume with composition change slope around x = 1.0–1.2 (the linear behaviour in lattice parameters expected from Vegard’s Law is shown by the dotted line). The deviation from Vegard’s Law is also evident in the composition dependence of the axial ratio *c*/*a* in Fig. 1.

The change in slope discerned around x = 1.0–1.2 is likely to be related to the change in magnetic ordering of the Mn-sublattice as in discussion of the neutron diffraction results below. At room temperature the x = 1.2 compound exhibits a mixture of ferromagnetic and antiferromagnetic states while compounds with x > 1.2 are purely antiferromagnetic. Similar tendencies in the composition dependence of the lattice constants have been detected in the related PrMn_2_Ge_2-x_Si_x_ system^25^,^26^. As is well known and as indicated above (see also Fig. 1), RMn_2_X_2_ (X = Ge or Si) compounds exhibit different magnetic behaviours around two critical values of the lattice parameter^9^. The bond lengths between different sites have also been calculated with the BLOKJE program^27^ using the structural and positional parameters and the 12-coordinate metallic radii of 1.81 Å, 1.35 Å, 1.37 Å and 1.32 Å for Ce, Mn, Ge and Si, respectively. It was found that the Mn–Mn intralayer distance at room temperature decreased from *d*_Mn-Mn_ = 2.93 Å at x = 0 to *d*_Mn-Mn_ = 2.83 Å at x = 2.0. Moreover if we assume that the contraction of the unit cell volume due to Si substitution (i.e. causing an effective chemical pressure compared with the reference CeMn_2_Ge_2_ compound) is equivalent to the influence of external pressure, the corresponding pressures can be derived to be around 43.6 kbar and 128.0 kbar for CeMn_2_GeSi and CeMn_2_Si_2_ respectively (details shown in Figure S2 of the Supplementary Material). The chemical pressure values were calculated from the Murnaghan equation:


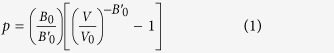


where *B*_0_ is the isothermal bulk modulus, *B*′_0_ its pressure derivative and *V*_0_ and *V* are the volume at ambient pressure and pressure *p* respectively. The range of pressure values for CeMn_2_GeSi and CeMn_2_Si_2_ was estimated from calculations based on the modulus values *B*_0_ = 819 kbar and *B*′_0_ = 4.0 for LaMn_2_Si_2_^13^ and *B*_0_ = 867 kbar and *B*′_0_ = 5.1 for CeNi_2_Ge_2_^28^ (see Figure S2; it should be noted that both LaMn_2_Si_2_ and CeNi_2_Ge_2_ are isostructural with the CeMn_2_Ge_2-x_Si_x_ compounds).

**Figure 2:**
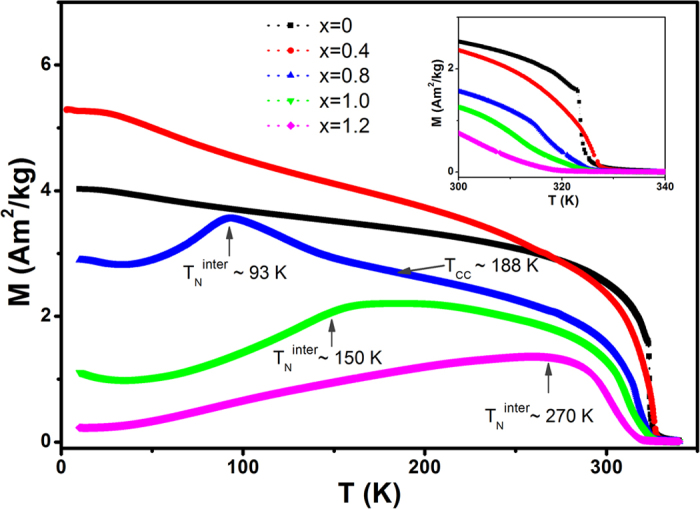
Temperature dependence of magnetization of CeMn_2_Ge_2-x_Si_2_ compounds (*x* = 0.0–2.0) as measured in a field of 0.01 T. The inset shows the magnetization in the region of T_C_^inter^.

Figure 2 shows the temperature dependence of the magnetization of CeMn_2_Ge_2-x_Si_x_ as measured in a field of 0.01 T over the temperature range of 5–340 K. Differential scanning calorimetry measurements have been used to check for possible phase transitions in the temperature region from 340 K to 550 K (details are shown in Figure S3). CeMn_2_Ge_2-x_Si_x_ compounds reveal up to four magnetic transitions: T_N_^inter^ – the Néel temperature associated with the onset of the axial component of antiferromagnetism; T_CC_ – the ferromagnetic critical temperature for incommensurate canted ferromagnetism; T_C_^inter^ – the Curie temperature of the axial component of ferromagnetism and T_N_^intra^, the Néel temperature for planar antiferromagnetism. Detailed definitions of the related magnetic structures are shown in Figure S1 with details of the magnetic structures discussed below in the neutron diffraction section. Figure 2 demonstrates that below 340 K the magnetic phase transition temperatures change with the Si concentration as expected, the T_C_^inter^ (magnetic phase transition temperature from interlayer antiferromagnetic AFl to canted ferromagnetic Fmc) was found to decrease from T_C_^inter^ ~ 320 K for x = 0.4 to T_C_^inter^ ~ 305 K for x = 1.2 (Fig. 2; inset). This behaviour may be related to the change in electronic environment as replacing Ge (3*d*^10^4*s*^2^4*p*^2^) by Si (3*s*^2^3*p*^2^) is expected to influence the magnetic structures of the CeMn_2_Ge_2-x_Si_x_ compounds and in addition to contracting the unit cell. This is supported by Density Functional Theory calculations for RMn_2_Ge_2_ (R = Y or Ca) compounds^29^ which indicate that to a large extent, the magnetic moment is determined mainly by the interatomic Mn–Mn distances, while the interstitial electron density contributes to the change in magnetic structures.

### Neutron diffraction; Magnetic structures

A set of neutron powder diffraction patterns was obtained for CeMn_2_Ge_2-x_Si_x_ compounds (x = 0.0–2.0) over the temperature range 4–450 K. Rietveld refinements were carried out on all patterns using the FULLPROF program package^24^ which allows us to derive the structural and magnetic parameters. As explained fully in related articles^8^,^19^,^25^,^30^, the specific location of Mn atoms on the 4*d* site in the ThCr_2_Si_2_ structure (space group *I4/mmm*) allows ready identification of various magnetic structures from key indicators in the neutron diffraction patterns as follows:

1. Ferromagnetic ordering of the Mn atoms—*hkl* reflections with *h* + *k* = 2*n* and *l* = 2*n* (e.g. (112), (200) reflections).
2. Antiferromagnetic ordering of the Mn atoms within the (001) planes—reflections with *h* + *k* = 2*n* + 1 (e.g. (101), (103) reflections).
3. Collinear antiferromagnetic structure between adjacent Mn planes—reflections with *h* + *k* + *l* = 2*n* + 1 (e.g. (111), (113) reflections).
4. Ferromagnetic mixed incommensurate structure (Fmi) of wavevector (0; 0; *q*_*z*_)—satellite reflections with h + k = 2n + 1 (e.g. (101), (103)).

**Figure 3:**
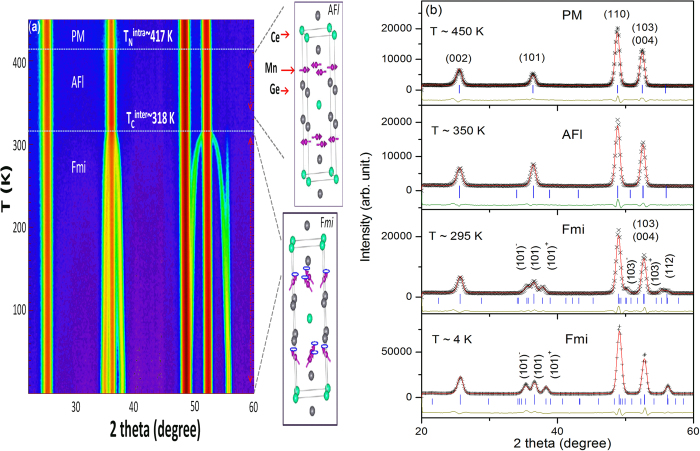
(**a**) Thermal contour plot of CeMn_2_Ge_2_ neutron diffraction measurements over the range of 4–450 K. The AFl and Fmi magnetic structures of CeMn_2_Ge_2_ are also shown; (**b**) Neutron diffraction patterns and Rietveld refinements for CeMn_2_Ge_2_ in different magnetic states at 450 K, 350 K, 295 K, and 4 K (λ = 2.4179 Å, Wombat diffractometer, OPAL).

The neutron diffraction thermal contour plot for CeMn_2_Ge_2_ from 4–450 K shown in Fig. 3(a), covers the various magnetic regions indicated by the magnetic measurements of Fig. 2 and Figure S3. Refinement of the 450 K neutron diffraction pattern confirms that CeMn_2_Ge_2_ is paramagnetic. At 350 K (i.e. below T_N_^intra^ ~ 417 K; Fig. 3(b)) the intensity of the (101) reflection increases and, consistent with neutron diffraction condition (2) above, CeMn_2_Ge_2_ exhibits the AFl structure. The appearance of the (112) peak at 295 K - below the transition temperature T_C_^inter^ (T_CC_) ~ 318 K - together with the presence of satellite peaks (101)^+^, (101)^−^ and (103)^+^, (103)^−^ in the patterns and noting condition (4) above, demonstrates that CeMn_2_Ge_2_ has the ferromagnetic mixed incommensurate (Fmi) magnetic structure below this transition temperature. The Fmi magnetic structure is found to persist with decrease in temperature to T ~ 4 K as confirmed by the absence of change in the intensities of the (101)^+^ and (101)^−^ reflections. Compared with the model reported by Fernandez-Baca *et al.*^18^ using the (1 0 1-q_z_) vector, we found that the vector (0 0 q_z_; q_z_ ~ 0.31) not only generates the (101)^−^/(101)^+^ and (103)^−^/(103)^+^ satellite peaks in their correct positions, but also results in an excellent fit of the magnetic satellites overall. Thus we conclude that our data for the Fmi magnetic structure are best described by the vector (0 0 q_z_); this is the same magnetic structure reported for LaMn_2_Ge_2_ by Venturini *et al.*^31^ and for RMn_2_Ge_2_ with R = Ce, Pr and Nd by Welter *et al.*^19^.

**Figure 4:**
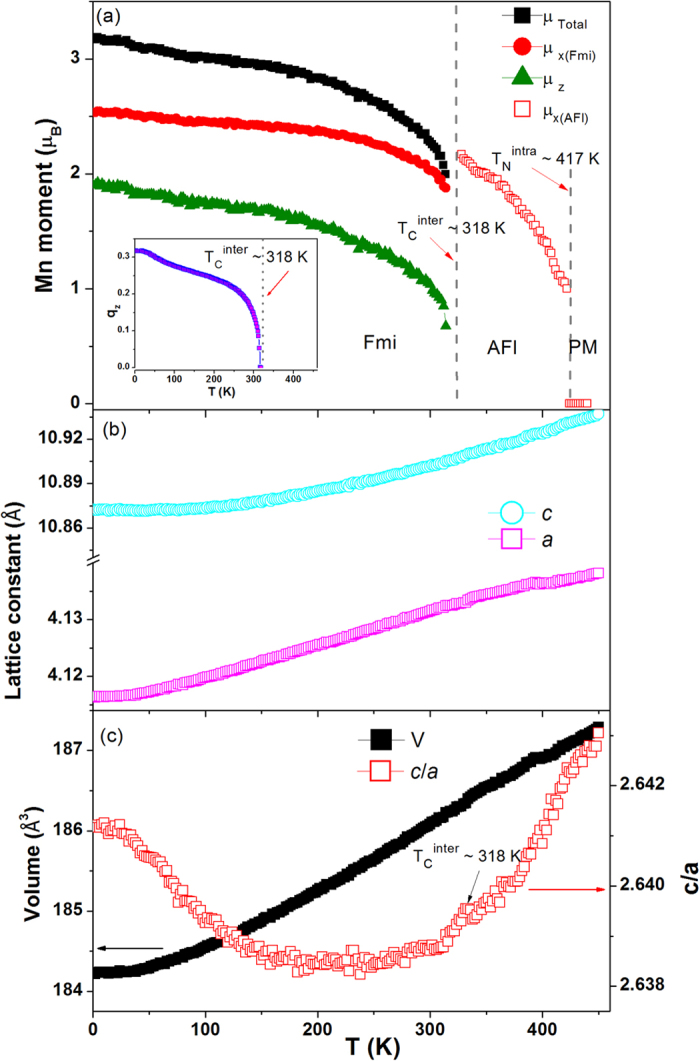
Structural and magnetic parameters for CeMn_2_Ge_2_ as derived from refinements of the neutron diffraction patterns of Fig. 3: (**a**) Temperature dependences of the magnetic moment and propagation vector *q*_*z*_ for CeMn_2_Ge_2_ (inset). T_N_^intra^ and T_C_^inter^ are denoted by arrows with the dotted lines delineating the paramagnetic (PM), antiferromagnetic (AFl-type) and ferromagnetic mixed incommensurate (Fmi) regions. (**b**) Temperature dependences of lattice parameters *a* and *c* and (**c**) unit cell volume and axial ratio *c*/*a*.

For CeMn_2_Ge_2_ the values of the magnetic moments at 4 K (see Table 1) are derived to be μ_Total_ ~ 3.16 μ_B_, μ_ab_ ~ 2.53 μ_B_ and μ_c_ ~ 1.9 μ_B_ with the magnitude of the propagation vector *q*_*z*_ ~ 0.317 (Fig. 4(a)). The propagation vector is found to decrease with increasing Si concentration with *q*_*z*_ ~ 0.276 for CeMn_2_Ge_1.6_Si_0.4_ at 4 K (Table 1; detailed results for CeMn_2_Ge_1.6_Si_0.4_ can be found in Figure S4). This decrease in *q*_*z*_ on replacement of Ge with Si in CeMn_2_Ge_2_ is similar to that detected on replacement of Mn with Fe^32^ and Pr with Y or Lu^9^,^33^ in PrMn_2_Ge_2_. The temperature dependences of the structural and magnetic parameters of CeMn_2_Ge_2_ as derived from the refinements are shown in Fig. 4(a–c). Temperature dependence of the *c*/*a* ratio reveals a significant change around T_C_^inter^ ~ 318 K (Fig. 4(c)). This phenomenon indicates that strong coupling occurs between the magnetism and the crystal lattice in the presence of a *c*-axis component of the Mn moment; this behaviour is similar to that of the PrMn_2-x_Fe_x_Ge_2_ system^32^, where the presence of the interlayer Mn–Mn interactions rather than the intralayer Mn–Mn interactions play the major role in the anomalous thermal expansion observed at the magnetic transition in these layered systems.

**Figure 5:**
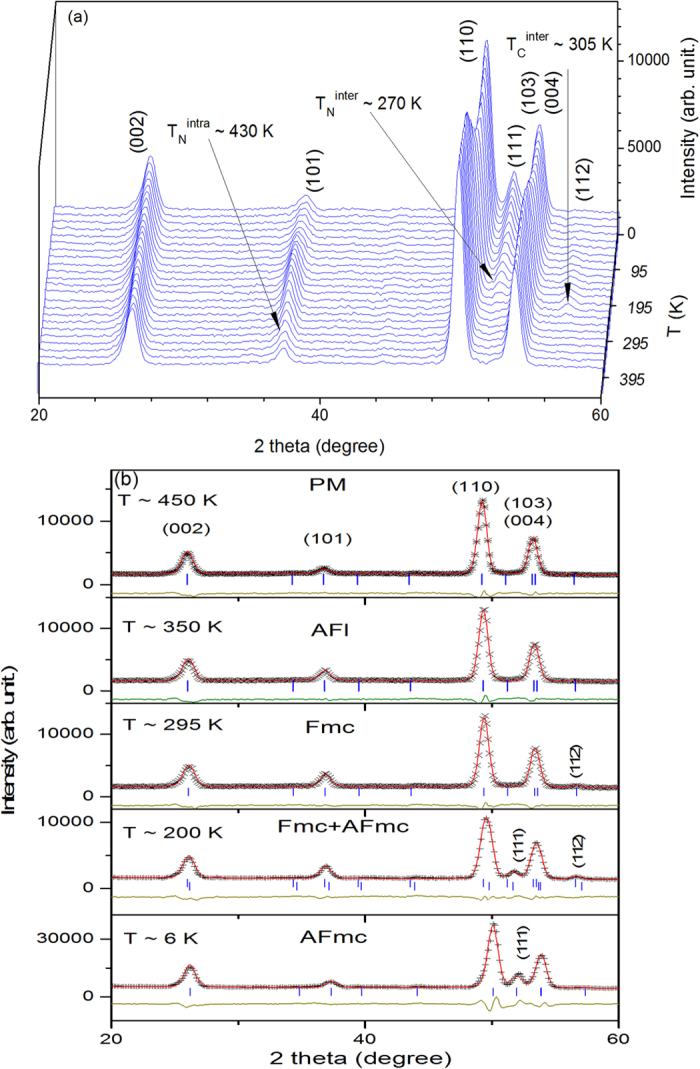
(**a**) Neutron diffraction patterns for CeMn_2_Ge_0.8_Si_1.2_ over the temperature range 6–450 K and (**b**) Rietveld refinements for CeMn_2_Ge_0.8_Si_1.2_ at 450 K, 350 K, 295 K, 200 K and 6 K (λ = 2.4118 Å , Wombat diffractometer, OPAL).

Reflecting the changes in magnetisation with temperature for the CeMn_2_Ge_1.0_Si_1.0_ and CeMn_2_Ge_0.8_Si_1.2_ compounds (Fig. 2), the neutron diffraction patterns of CeMn_2_Ge_1.0_Si_1.0_ and CeMn_2_Ge_0.8_Si_1.2_ were also found to exhibit interesting behaviour. Neutron diffraction patterns were collected for both compounds over the temperature range 6–450 K with temperature steps of around 6 K in order to obtain detailed information around the phase transitions. The set of neutron diffraction patterns for CeMn_2_Ge_0.8_Si_1.2_ are shown as an example in Fig. 5(a) with the refinement results at selected temperatures listed in Table 2.

Among features noted for CeMn_2_Ge_0.8_Si_1.2_ are that the intensity of the (002) nuclear reflection remains effectively unchanged over the whole temperature range and the absence of satellite peak (101)^+^, (101)^−^ and (103)^+^, (103) compared with CeMn_2_Ge_2_ and CeMn_2_Ge_1.6_Si_0.4_. Magnetic phase transitions at T_N_^intra^ ~ 430 K; T_C_^inter^ ~ 305 K and T_N_^inter^ ~ 270 K are clearly indicated by changes in the intensities of the (101), (111) and (112) reflections due to magnetic scattering, with details shown in Supplementary Figure S5. Figure S5 also indicates the temperature T* ~ 31 K below which only the AFmc structure exists in CeMn_2_Ge_0.8_Si_1.2_. This is evident on comparison of the diffraction patterns in Fig. 5(b) for CeMn_2_Ge_0.8_Si_1.2_ at 200 K (mixed Fmc and AFmc states) with the diffraction pattern of the AFmc state at 6 K. According to neutron diffraction condition (3), the appearance of the (111) reflection peak below ~ 270 K at T ~ 200 K in Fig. 5(b) indicates the formation of the AFmc state while, based on neutron diffraction condition (2), the fact that the (112) reflection remains indicates that the Fmc state still exists at the same temperature as the mixed magnetic states behaviour. However the mixture of the Fmc and AFmc magnetic states starts to disappear when cooling to T* < 31 K as the (112) reflection is absent and the (111) peak intensity remains unchanged; this behaviour indicates the occurrence of the AFmc state (see Fig. 5(b) for CeMn_2_Ge_0.8_Si_1.2_ at T ~ 6 K).

**Figure 6:**
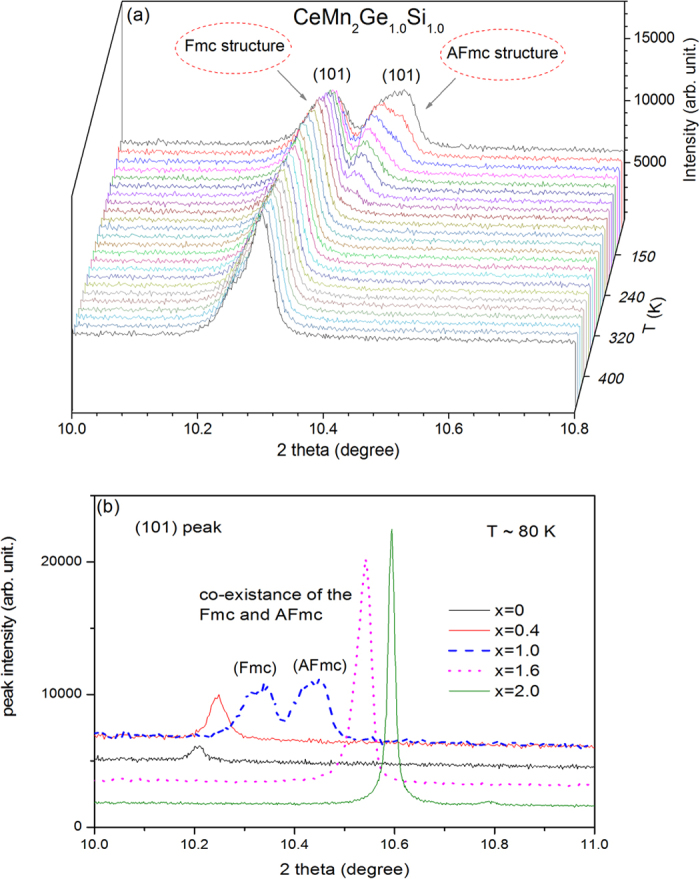
(**a**) X-ray diffraction patterns over the range of 80–450 K for CeMn_2_Ge_1.0_Si_1.0_ in the 2 ⊖ region around the (101) peak position, and (**b**) comparison of the reflections observed around the (101) peak position for CeMn_2_Ge_2-x_Si_x_ compounds of Si concentrations x = 0.0, x = 0.4, x = 1.0, x = 1.6 and x = 2.0 at 80 K (λ = 0.6887 Å, Powder Diffract, Australian Synchrotron).

Direct evidence for the coexistence of two magnetic phases in the CeMn_2_Ge_1.0_Si_1.0_ sample was obtained using high resolution synchrotron X-ray diffraction; Fig. 6(a) indicates two distinct (101) reflections from ~80 K to ~180 K with a single (101) reflection observed above ~180 K. These structural features are entirely consistent with the occurrence of mixed structures of the ferromagnetic Fmc and antiferromagnetic AFmc states below ~180 K. This behaviour was confirmed by comparison of the (101) peak for various CeMn_2_Ge_2-x_Si_x_ compounds at 80 K (Fig. 6(b)). Comparison of these high resolution X-ray diffraction patterns reveals that only the CeMn_2_Ge_1.0_Si_1.0_ sample exhibits mixed (101)-type peaks at 80 K compared with the single peak behaviour exhibited by CeMn_2_Ge_2-x_Si_x_ compounds of Si concentrations x = 0.0, x = 0.4, x = 1.6 and x = 2.0. In addition co-existence of the (111) and (112) peaks as observed in the neutron diffraction patterns of CeMn_2_Ge_0.8_Si_1.2_ below T_N_^inter^ ~ 270 K (Fig. 5 at T ~ 200 K), indicates co-existence of two magnetic states corresponding to the ferromagnetic Fmc and antiferromagnetic AFmc states. This behaviour is similar to the co-existence of magnetic phases first reported in La_0.8_Y_0.2_Mn_2_Si_2_^34^. The Rietveld refinements also confirm that the unit cell for the AFmc phase is smaller than the unit cell for the Fmc phase (e.g. for CeMn_2_Ge_0.8_Si_1.2_ at T = 200 K the unit cell volume V ~ 178.6 Å^3^ for Fmc while V ~ 174.5 Å^3^ for AFmc as listed in Table 2). This behaviour agrees well with the other re-entrant ferromagnetism systems^20^,^25^,^35^, with around 0.3% contraction of the unit cell observed when the magnetic state changes at T_N_^inter^ from Fmc to AFmc in SmMn_2_Ge_2_^35^.

It is recognised that chemical distribution is expected on the mixed lattice site^3^,^7^,^29^ in pseudo-ternaries. Although we can rule out long-range ordering of Si and Ge which would have been obvious due to substantial contrast in their neutron scattering lengths (4.15 fm and 8.19 fm, respectively), it is difficult to establish from neutron diffraction studies whether the distribution is completely random or some short-range ordering is present. Similar to our findings for the PrMn_2_Ge_2-x_Si_x_ system^29^, our high resolution synchrotron data of a series of CeMn_2_Ge_2-x_Si_x_ samples (x = 0, 1.0, 1.6 and 2.0), at 450 K in the paramagnetic state (shown in Supplementary Figure S6) shows that the limiting compounds - CeMn_2_Si_2_ (of full width at half maximum for the (101) reflection of FWHM = 0.0103°) and CeMn_2_Ge_2_ (FWHM = 0.0305° for the (101) reflection), - have peak widths which are narrower than for samples with mixed Si and Ge (*e.g.* FWHM = 0.0361° and FWHM = 0.0587° for the (101) reflection of CeMn_2_Ge_0.4_Si_1.6_ and CeMn_2_Ge_1_Si_1_ respectively). These experimental findings demonstrate the occurrence of local stoichiometry fluctuations, particularly for the x = 1.0 sample. Rietveld analyses of high resolution synchrotron data for the CeMn_2_Ge_1_Si_1_ sample in the paramagnetic state at 450 K, shows the presence of two phases with different concentrations: one is Si rich (53.5% phase fraction with a = 4.1006(8) Å and c = 10.7483(9) Å) and another is Ge rich (46.5% phase fraction with 4.1160(8) Å and c = 10.8091(9) Å). While the presence of these two phases due to stoichiometry fluctuations results in a width of the (101) peak at 450 K of around ~0.1°, the separation of the two (101) peaks is significantly larger (~0.26°; see 80 K synchrotron data of Fig. 6(b)) due to the presence of the two magnetic phases AFmc and Fmc. The average lattice strain (δ*a*/*a*) values determined from Rietveld refinements of the high resolution synchrotron data of the CeMn_2_Ge_2-x_Si_x_ compounds at 450 K in the paramagnetic state are: 0.012, 0.016, 0.019, 0.014 and 0.005 for x = 0, 0.4, 1.0, 1.6 and 2.0 respectively (as shown in Figure S6). The variation in strain values with composition for CeMn_2_Ge_2-x_Si_x_ displays similar trends to those observed in the PrMn_2_Ge_2-x_Si_x_ system^29^.

**Figure 7:**
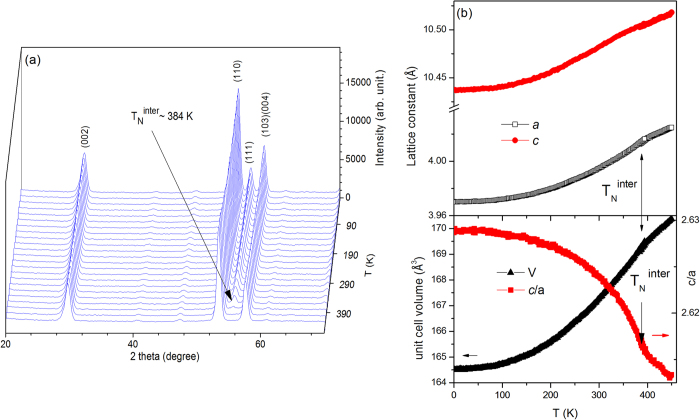
(**a**) Neutron diffraction patterns for CeMn_2_Si_2_ over the range of 6–450 K (λ = 2.4118 Å) and (**b**) Temperature dependences of the magnetic moment of CeMn_2_Si_2_ and the (111) peak (inset). (**c**) Temperature dependences of the lattice parameters *a* and *c* together with the unit cell volume, V, and axial ratio *c*/*a* for CeMn_2_Si_2_ as determined from Rietveld refinements of the neutron diffraction patterns.

Details about the phase transitions in CeMn_2_Si_2_ over the temperature range 6–450 K were determined from neutron diffraction patterns obtained at temperature step intervals of 2 K (Fig. 7(a)). Rietveld refinements of the neutron diffraction pattern at 450 K confirm that CeMn_2_Si_2_ has the ThCr_2_Si_2_ structure as expected. The absence of magnetic scattering above T_N_^intra^ ∼ 384 K in reflections such as (101), (111) and (112) is consistent with a paramagnetic (PM) state (see e.g. the disordered magnetic states (PM) observed in EuMn_2_Si_2_^36^ and LaPrMn_2_Si_2_^37^. Below T_N_^intra^ ∼ 84 K, CeMn_2_Si_2_ is found to exhibit the antiferromagnetic interlayer coupling structure (AFil) down to the experimental base temperature of T ∼ 4 K (Fig. 7(c) and Table 1) in agreement with the findings of previous studies^18^. The AFil structure — a collinear antiferromagnetic structure between adjacent Mn planes in a + − + − sequence along the *c*-axis as depicted in Figure S1 — is indicated by the magnetic scattering observed at the (111), (113) and (201) reflections (extinction rules *h* + *k* = 2*n* and *h* + *k* + *l* = 2*n* + 1) in agreement with those reported by Dincer *et al.*^37^. Figure 7(b) shows the temperature dependences (4–450 K) of the lattice parameters and *c*/*a* axial ratio as determined from Rietveld refinements of the neutron diffraction patterns. Both the *a* and *c* values exhibit a monotonic decrease with temperature in the region of the paramagnetic to interlayer antiferromagnetic transition. Furthermore, Fig. 7(b) reveals a significant change in the temperature dependence of the *c*/*a* ratio around T_N_^inter^ ∼ 384 K; this behaviour indicates strong coupling between the magnetism and the crystal lattice in the presence of a *c*-axis component of the Mn moment. As discussed recently^38^, strong magnetostructural coupling leads to a large structural entropy change around the magnetic phase transition, thereby contributing to the total entropy change around the magnetic phase transition.

### Magnetocaloric effect and critical exponent analysis

The magnetic entropy change, −∆S_M_, has been determined for the set of CeMn_2_Ge_2-x_Si_x_ compounds (x = 0.0–2.0) from their magnetization curves as functions of temperature and magnetic field (∆B = 0–5 T) in the region around the ferromagnetic transition T_C_^inter^. The set of −∆S_M_ values was determined by applying the standard Maxwell relation^39^:


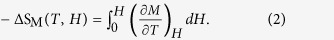


**Figure 8:**
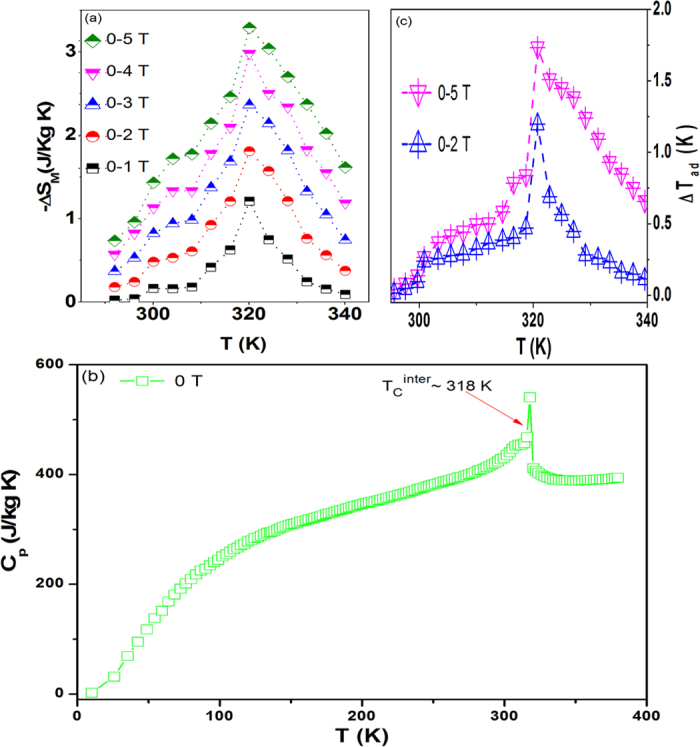
(**a**) Temperature dependence of the isothermal magnetic entropy change −∆S_M_ for CeMn_2_Ge_2_ in the region around the ferromagnetic transition T_C_^inter^ as determined from magnetisation measurements. (**b**) The heat capacity of CeMn_2_Ge_2_ as measured over the temperature range 10–340 K with no magnetic fields *B* = 0 T; (**c**) The adiabatic temperature change,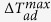, for the CeMn_2_Ge_2_ compound.

As shown by the curves of Fig. 8(a), the −∆S_M_ peak gradually broadens towards higher temperatures with increasing magnetic field (from ∆B = 0–5 T), behaviour characteristic of a second order magnetic transition. The changes in magnetic entropy for the set of CeMn_2_Ge_2-x_Si_x_ compounds (x = 0, 0.4 and 0.8) have been derived to be 3.21 J kg^−1^ K^−1^, 2.86 J kg^−1^ K^−1^ and 2.67 J kg^−1^ K^−1^, respectively (field change ∆B = 0–5 T) around T_C_^Inter^. CeMn_2_Ge_2-x_Si_x_ compounds exhibit moderate isothermal magnetic entropy accompanied with a second-order phase transition around room temperature comparable with other rare earth intermetallic compound in the RMn_2_X_2_ series. For example, Dincer and Elerman^40^ obtained maximum entropy values in the approximate range −∆S_M _~ 2–3 J kg^−1^ K^−1^ (∆B = 0–5 T) around the Curie temperatures T_C_^inter^ ~ 300–320 K for re-entrant SmMn_2-x_Fe_x_Ge2 (x = 0.05, 0.10) and SmMn_2-x_Co_x_Ge_2_ (x = 0.05, 0.15) compounds. The present set of entropy values are also similar to other compound such as Ho_2_Fe_15_Mn_2_ 41 (−∆S_M_ = 2.7 J kg^−1^ K^−1^ at 302 K) and Er_2_Fe_17_ 42 (−∆S_M _= 3.6 J kg^−1^ K^−1^ at 300 K).

The magnetic entropy change, −∆S_M_ (*T,* *B*) has also been derived from heat calorimetric measurements of the field dependence of the heat capacity using the expression^43^,^44^,^45^:


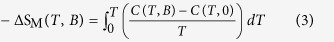


where *C(T,B)* and *C(T,0)* are the values of the heat capacity measured in field *B* and zero field, respectively. The corresponding adiabatic temperature change, ∆*T*_*ad*_ can be evaluated from −∆S_M_ (*T,* *B*) and the zero field heat capacity data as:


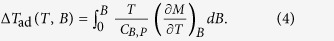


Figure 8(b) show the set of heat capacity measurement obtained for CeMn_2_Ge_2_ with B = 0 T. The related peak in specific heat around T_C_^inter^ ~ 318 K was found to decrease with increasing magnetic field. The ∆*T*_ad_ values derived from the specific heat data using equation (4) are shown in Fig. 8(c). The peak value of the adiabatic temperature change is found to be 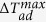 = 1.7 K for ∆*B* = 0–5 T. Within experimental errors^23^,^46^,^47^, the maximum magnetic entropy change for CeMn_2_Ge_2_ as determined from the heat capacity measurements of −∆S_M_^max^ ~ 2.9 J kg^−1^ K^−1^ agrees well with the maximum entropy change −∆S_M_^max^ ~ 3.2 J kg^−1^ K^−1^ determined from the magnetic measurements using the Maxwell relation.

**Figure 9:**
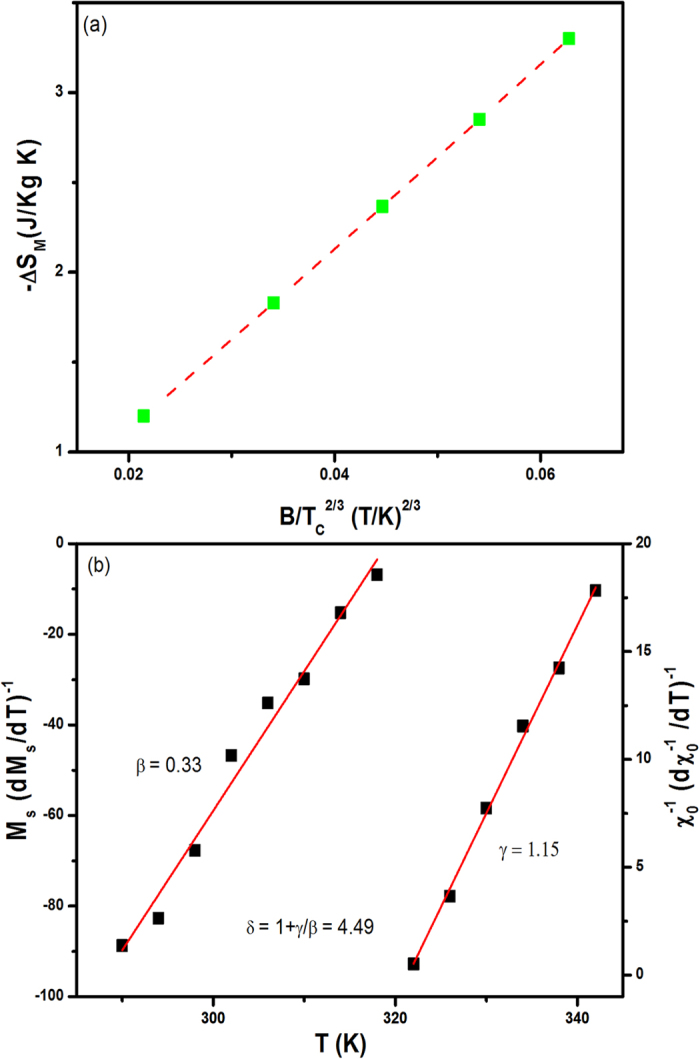
(**a**) Dependence of −∆S_M_ (peak value of the magnetic entropy change at different B values) on the parameter (B/T_C_)^2/3^ for CeMn_2_Ge_2_ compound and (**b**) Kouvel–Fisher plots of 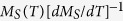 (left scale) and 
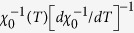
 (right scale) versus temperature. The lines are fits to the data around T_C_ as discussed in the text with fits leading to the critical exponent values.

Critical exponent analysis: Mean-field theory predicts that in the vicinity of second-order phase transitions, −∆S_M_ is proportional to (μ_0_H/T_C_)^2/3^ [Refs. 48, 49]. Figure 9(a) shows a graph of −∆S_M_ as a function of (B/T_C_)^2/3^ in the region around the magnetic transition at T_C_^inter^ ~ 318 K. The linear fit to the data in Fig. 9(a) clearly demonstrates that the relationship −∆S_M_ ∝ (B/T_C_)^2/3^ is valid around the transition at T_C_^inter^ for CeMn_2_Ge_2_. According to the conventional static scaling law, the critical properties of a second-order magnetic transition can be described by critical exponents β, γ and δ derived from magnetization measurements around the transition temperature. On applying these standard approaches, as shown by the fits to the Kouvel–Fisher plots of 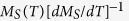
and 
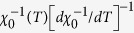
versus temperature in Fig. 9(b), the critical exponents around T_C_ in CeMn_2_Ge_2_ have been determined to be β = 0.33 ± 0.03 and γ = 1.15±0.22. Hence, on applying the relationship δ = 1 + γ/β, with β = 0.33, γ = 1.15, the critical exponent δ = 1 + γ/β = 4.49 ± 0.25. The critical exponents derived from the analyses are similar to the theoretical values - β = 0.365, γ = 1.386 and δ = 4.80 - based on the three-dimensional Heisenberg model corresponding to short range interactions^50^. Thus, the critical behaviour analysis in the vicinity of T_C_^inter^ indicates that the magnetism of the CeMn_2_Ge_2_ compound is governed by short range interactions.

## Discussion

The total magnetic moment of CeMn_2_Ge_2-x_Si_x_ compounds at base temperature ~5 K is found to decrease with increasing Si content (see Table 1). This behaviour indicates that contraction of the unit cell leads to a reduction in the Mn moment value (e.g. μ_Total_ ~ 3.16 μ_B_ for x = 0.0 and μ_Total_ ~ 2.02 μ_B_ for x = 2.0) and agrees well with the tendency detected for both the LaMn_2_Ge_2_ and LaMn_2_Si_2_ systems where μ_Total_ is found to decrease with decrease in the lattice parameter *a*^51^. First principles calculations on LaMn_2_Si_2_ and LaMn_2_Ge_2_^51^ suggest that the reduction of Mn moments in LaMn_2_Si_2_ (*a* = 4.11 Å) compared with LaMn_2_Ge_2_ (*a* = 4.19 Å) depends primarily on the Mn–Mn distances (stronger Mn–Mn hybridization due to shorter Mn–Mn distance leads to a smaller local Mn moment^19^). However the larger hybridization strength of Si–Mn in LaMn_2_Si_2_ than Ge–Mn in LaMn_2_Ge_2_ also plays a role. In the present study, the reduction of Mn moments in the CeMn_2_Ge_2-x_Si_x_ compounds with increasing Si content can be ascribed to these two factors: (1) decrease of the Mn–Mn spacing (at room temperature ~300 K, *d*_Mn-Mn_ = 2.93 Å at CeMn_2_Ge_2_ to *d*_Mn-Mn_ = 2.83 Å at CeMn_2_Si_2_) and (2) increase of Si–Mn hybridization strengths compared with Ge–Mn hybridization^24^, similar behaviour to that of PrMn_2_Ge_2-x_Si_x_^25^.

The temperature dependence of the CeMn_2_Ge_2-x_Si_x_ lattice parameters demonstrates an anomaly in thermal expansion from low temperature to high temperature as indicated by the *c*/*a* ratio (for example CeMn_2_Ge_2_ in Fig. 4(c) and CeMn_2_Si_2_ in Fig. 7(b)) and is accompanied with the appearance of interlayer Mn–Mn interactions. This behaviour agrees well with others system as PrMn_2-x_Fe_x_Ge_x_ and PrMn_2_Ge_2-x_Si_x_ where it is found that the interlayer Mn–Mn interactions rather than the intralayer Mn–Mn interactions, play the major role in the anomalous thermal expansion^30^.

**Figure 10:**
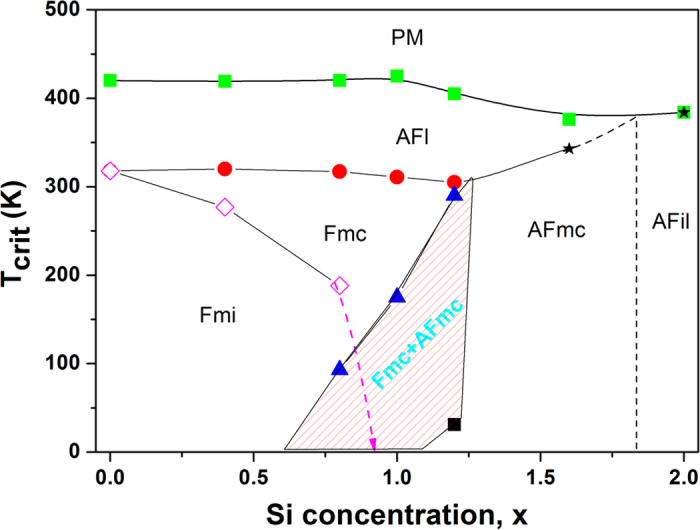
Magnetic phase diagram of CeMn_2_Ge_2-x_Si_x_ as a function of Si content. As discussed in the text, T_N_^intra^ (green squares) defines the transition from paramagnetism to intralayer antiferromagnetic ordering within the (001) Mn layers (AFl) (except x = 2.0 which exhibits AFil type antiferromagnetic order); T_C_^inter^ (red circles) defines the transition from AFl to a canted spin structure (Fmc); Tc/c (open pink diamond) defines the transformation temperature of the magnetic structure from Fmc to a conical configuration Fmi type, T_N_^inter^ (blue triangle) denotes the transition to the mixed region with co-existence of the antiferromagnetic canted structure AFmc and the Fmc structure. The dashed lines indicate trends in the data. As discussed in the text, the vertical dashed line located around Si concentration of x = 1.85 is used as a tentative guide to the boundary between the AFmc and AFil regions.

The present investigation of the magnetic and structural properties of a series of CeMn_2_Ge_2-x_Si_x_ compounds (x = 0.0, 0.4, 0.8, 1.0, 1.2, 1.6 and x = 2.0) have enabled us to construct the magnetic phase diagram for the CeMn_2_Ge_2-x_Si_x_ system as shown in Fig. 10 with more detail now available compared with previous studies^16^. The shaded region in Fig. 10 indicates the region of co-existence of the Fmc and AFmc phases as established here. The co-existence of different magnetic states at the same temperature is considered to be related to the non-random variation of site concentrations of Si and Ge, and depends sensitively on the Mn–Mn distances in this system; this in turn leads to differences in the local environments throughout the sample^7^.

As expected, the magnetic states at room temperature have been modified by Si substitution due to the contraction of the unit cell indicated in Fig. 1. Samples with x ≥ 1.4 where the lattice constant *a* is below *a*_*crit1*_ ~ 4.06 Å are antiferromagnetic at room temperature with no ferromagnetic order evident over the entire temperature range, whereas samples of Si content x ≤ 0.4 are ferromagnetic at room temperature. It is interesting to note that samples with Si concentrations in the range x ~ 1.0–1.2 correspond approximately to the region of co-existence of the AFmc and Fmc phases around room temperature as shown in Fig. 10. A smaller unit cell in an antiferromagnetic state than in a ferromagnetic state (as indicated by the deviation from linear behaviour in the composition dependence of the lattice constants at room temperature; Fig. 1), can be understood in terms of the difference in magnetic states at room temperature for these samples and in turn reflects the large contribution from magnetic effects to thermal expansion^30^.

The Si-rich compounds with x ≥ 1.85 have a relatively simple magnetic behaviour, transforming from paramagnetism at high temperature to AFil. By comparison, Ge-rich samples (x ~ 0.0) successively exhibit two magnetic states as AFl and Fmi when cooling from the high temperature paramagnetic phase. As depicted in Fig. 10 , the Fmi structure was eliminated for Si concentrations x ≥ 1.0, and co-existence of the AFmc and Fmc states is detected for CeMn_2_Ge_2-x_Si_x_ in the intermediate Si concentration range 0.6 < x < 1.25. Moreover, critical properties study on the second-order ferromagnetic transition of CeMn_2_Ge_2_ demonstrate that the magnetic interactions around T_C_^inter^ can be described with the three dimensional Heisenberg model corresponding to short range interactions. Overall this investigation has demonstrated that, as expected, the Si concentration plays the dominant role in tuning the magnetic structure and properties of the CeMn_2_Ge_2-x_Si_x_ compounds.

## Methods

CeMn_2_Ge_2-x_Si_x_ alloys with Si concentrations x = 0.0, 0.4, 0.8, 1.0, 1.2, 1.6 and x = 2.0 were prepared by using standard arc melting with high purity elements on a water-cooled Cu hearth under purified argon gas. The mass loss of Mn during melting was compensated for by adding 3% excess Mn. The ingots were melted five times to attain homogeneity and then annealed at 900 °C for one week in an evacuated quartz tube. The samples were characterized by high intensity X-ray powder diffraction (λ = 0.6887 Å; 80–450 K) carried out at the Australian Synchrotron. The magnetic properties were investigated over the temperature range 6–350 K using the vibrating sample magnetometer option of a Quantum Design 14 T physical properties measurement system (PPMS). All samples were investigated by differential scanning calorimetry (DSC) to check for possible phase transitions in the higher temperature range from 300 K to 450 K. Specific heat measurements were carried out from 10 K to 360 K with applied field 0 T, 1 T, 2 T and 5 T. Neutron diffraction patterns were collected over the temperature range 6–450 K to cover the temperature range over which magnetic transitions were observed. The neutron diffraction experiments were carried out on the Wombat diffractometer (high intensity diffractometer; with λ ~ 2.41 Å), OPAL, Australia.

## Additional Information

**How to cite this article**: Md Din, M. F. *et al.* Tuneable Magnetic Phase Transitions in Layered CeMn_2_Ge_2-x_Si_x_ Compounds. *Sci. Rep.* **5**, 11288; doi: 10.1038/srep11288 (2015).

## Supplementary Material

Supplementary Information

## Figures and Tables

**Table 1:** Structural and magnetic parameters derived from Rietveld refinements of the neutron diffraction patterns for CeMn_2_Ge_2-x_Si_x_ at 4 K and 6 K as indicated.

**Composition**	**CeMn_2_Ge_2_**	**CeMn_2_Ge_1.6_Si_0.4_**	**CeMn_2_Ge_0.8_Si_1.2_**	**CeMn_2_Ge_0.4_Si_1.6_**	**CeMn_2_Si_2_**
T (K)	4 K	4 K	6 K	6 K	4 K
Magnetic state	Fmi	Fmi	AFmc	AFmc	AFil
a (Å)	4.1165(4)	4.0992	4.0264	4.0049	3.9695
c (Å)	10.8723(6)	10.7942	10.6335	10.5501	10.4354
z_Ge_	0.3821	0.3819	0.3814	0.3795	0.3816
μ_ab_ (μ_B_)	2.53(3)	2.32	0.76	0.32	0
μ_c_ (μ_B_)	1.90(5)	1.81	2.00	2.09	2.01
Canting angle	53.1	52.0	20.8	8.7	0
μ_total_ (μ_B_)	3.17(7)	2.95	2.14	2.12	2.01
q_z_	0.317	0.276	–	–	–
R_wp_	5.81	7.41	9.52	5.55	7.50
R_exp_	1.18	1.22	1.23	1.06	1.49

The errors are shown for the CeMn_2_Ge_2_ data as an example.

**Table 2:** Structural and magnetic parameters derived from Rietveld refinements of the neutron diffraction patterns for CeMn_2_Ge_0.8_Si_1.2_.

**T (K)**	**6 K**	**200 K**	**200 K**	**295 K**	**350 K**	**450 K**
**Magnetic state**	**AFmc**	**54% AF*****mc***	**46% F*****mc***	**F*****mc***	**AF*****/***	**PM**
a (Å)	4.0264(5)	4.0482	4.0831	4.0801	4.0846	4.0918
c (Å)	10.6335(4)	10.6479	10.7110	10.6889	10.7005	10.7235
z_Ge_	0.3814	0.3818	0.3817	0.3814	0.3814	0.3814
μ_ab_ (μ_B_)	0.76(8)	0.76	2.04	1.48	1.13	–
μ_c_ (μ_B_)	2.00(6)	2.00	1.81	0.87	–	–
Canting angle	20.8	20.8	48.4	59.6	90	–
μ_total_ (μ_B_)	2.14(9)	2.44	2.72	1.72	1.13	–
R_wp_	9.52	7.18	7.18	6.14	6.67	6.52
R_exp_	1.23	2.20	2.20	2.19	2.19	2.17

The errors are shown for the 6 K data as an example.
