# Supplementary material for: Tuneable Magnetic Phase Transitions in Layered CeMn2Ge2-xSix Compounds
Source: Sci Rep. 2015 Jun 19;5:11288. doi: 10.1038/srep11288 (PMC4650650; doi:10.1038/srep11288)
Supplement: Supplementary Information [file srep11288-s1.pdf]

Supplementary Information for:

Tuneable Magnetic Phase Transitions in Layered $CeMn_2Ge_{2-x}Si_x$ Compounds

M. F. Md Din[1,4,*], J. L. Wang[1,2*], Z. X. Cheng[1], S. X. Dou[1], S. J. Kennedy[2] and S. J. Campbell[3]

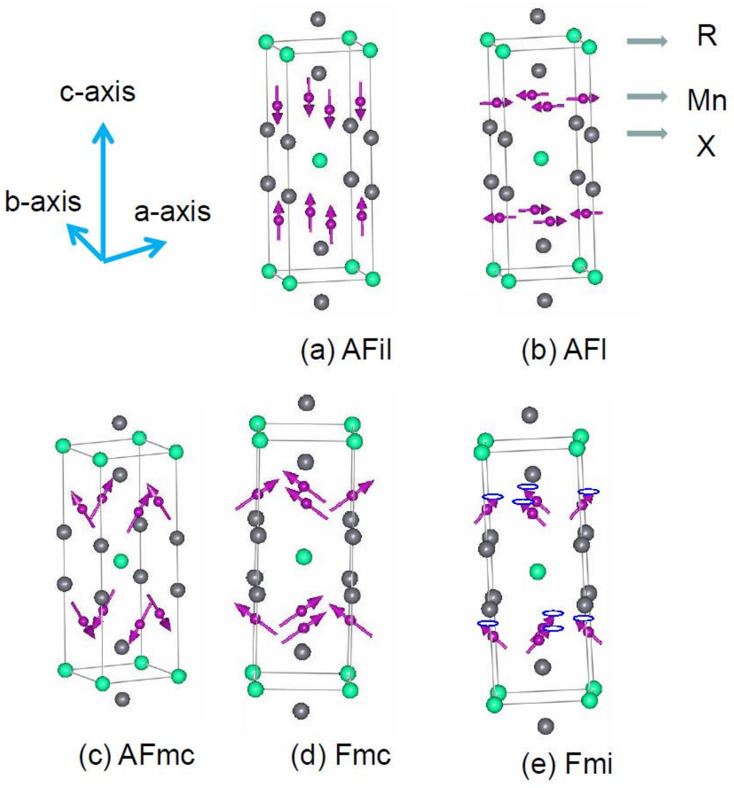

Figure S1 (Color online) Magnetic structures relevant to discussion of the $CeMn_2Ge_{2-x}Si_x$ compounds (R=Ce atoms—larger light circles; X=Ge or Si atoms—larger dark circles; Mn atoms—small circles). The different structure types are defined in [8, 9].

(a) AFil: No intralayer in-plane spin component and the interlayer coupling being antiferromagnetic. AFil is described with $I_P4/m'm'm'$ magnetic space group (Opechowski-Guccione #139.17.1195; basis (a, b, c; 1/4, 1/4, 1/4); active vector (0,0,1))

(b) AFl: No interlayer spin component and intralayer antiferromagnetic within *ab*-plane. AFl is described with the Im'mm magnetic space group (Opechowski-Guccione #71.3.623, basis (-b, a, c; 0,0,0); active vector (0,0,0)).

(c) AFmc: the antiferromagnetic mixed commensurate structure; characterized by the antiferromagnetic interplane coupling of the in-plane ferromagnetic components and by the commensurate ordering of the antiferromagnetic in-plane components. AFmc structure is described by the Pnnm’ magnetic space group (Opechowski-Guccione #58.4.474, basis (-a, c, b; 0,0,0); active vector (0,0,1)).

(d) Fmc: ferromagnetic mixed commensurate structure; characterized by the ferromagnetic interplane coupling of the in-plane ferromagnetic components and by the commensurate ordering of the antiferromagnetic in-plane components, Fmc structure is described by the Im’m2’ magnetic space group (Opechowski-Guccione #44.3.326, basis (b,c,a;0 0 0); active vector (0,0,0)).

(e) Fmi: ferromagnetic mixed incommensurate structures characterised by the ferromagnetic interplane coupling of the in-plane ferromagnetic components and by the incommensurate arrangement of the antiferromagnetic in-plane components.

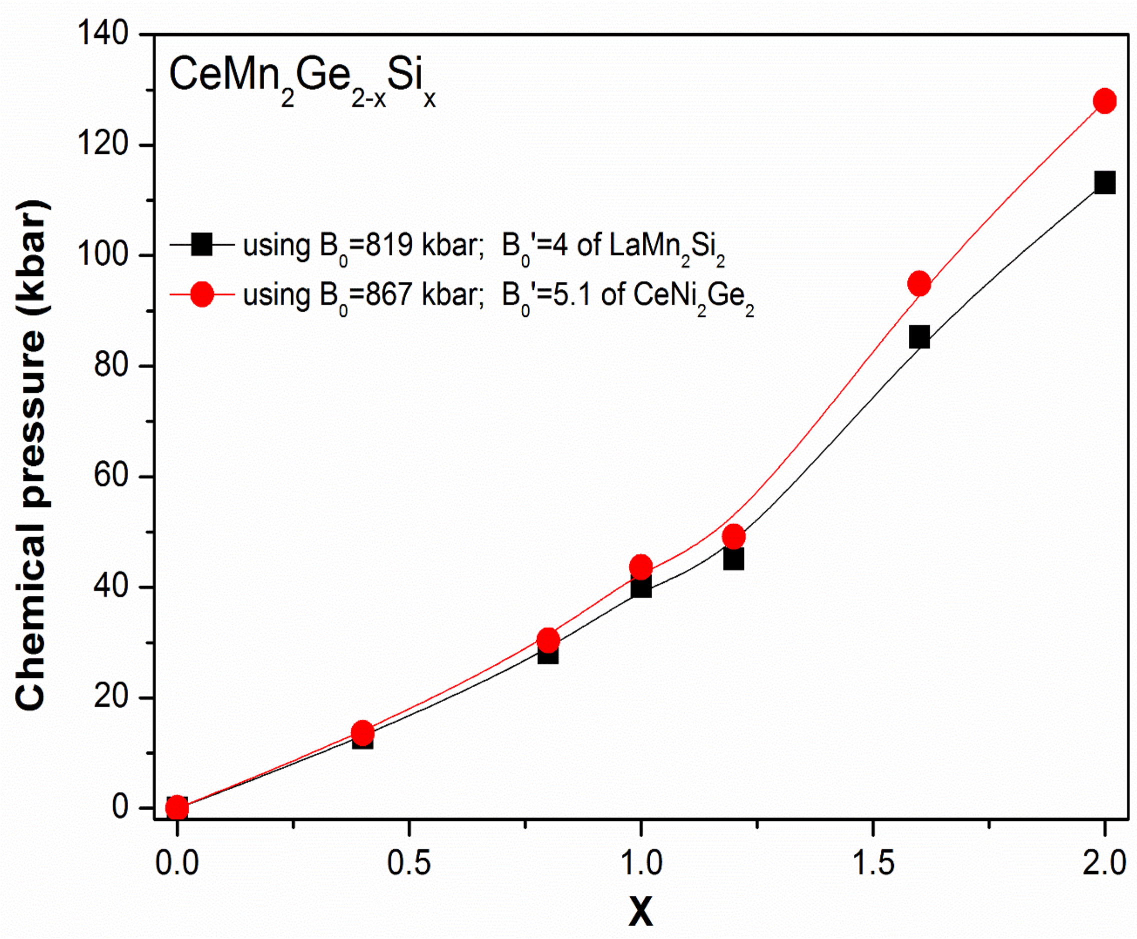

Figure S2 The calculated chemical pressures for $CeMn_2Ge_{2-x}Si_x$ based on the average values of B_0 and B_0' for the $LaMn_2Si_2$ [REF. 13] and $CeNi_2Ge_2$ [REF. 28] compounds using the Murnaghan equation. The graphs indicate clear trend in behaviour for $CeMn_2Ge_{2-x}Si_x$ compounds and their likely range of chemical pressure values.

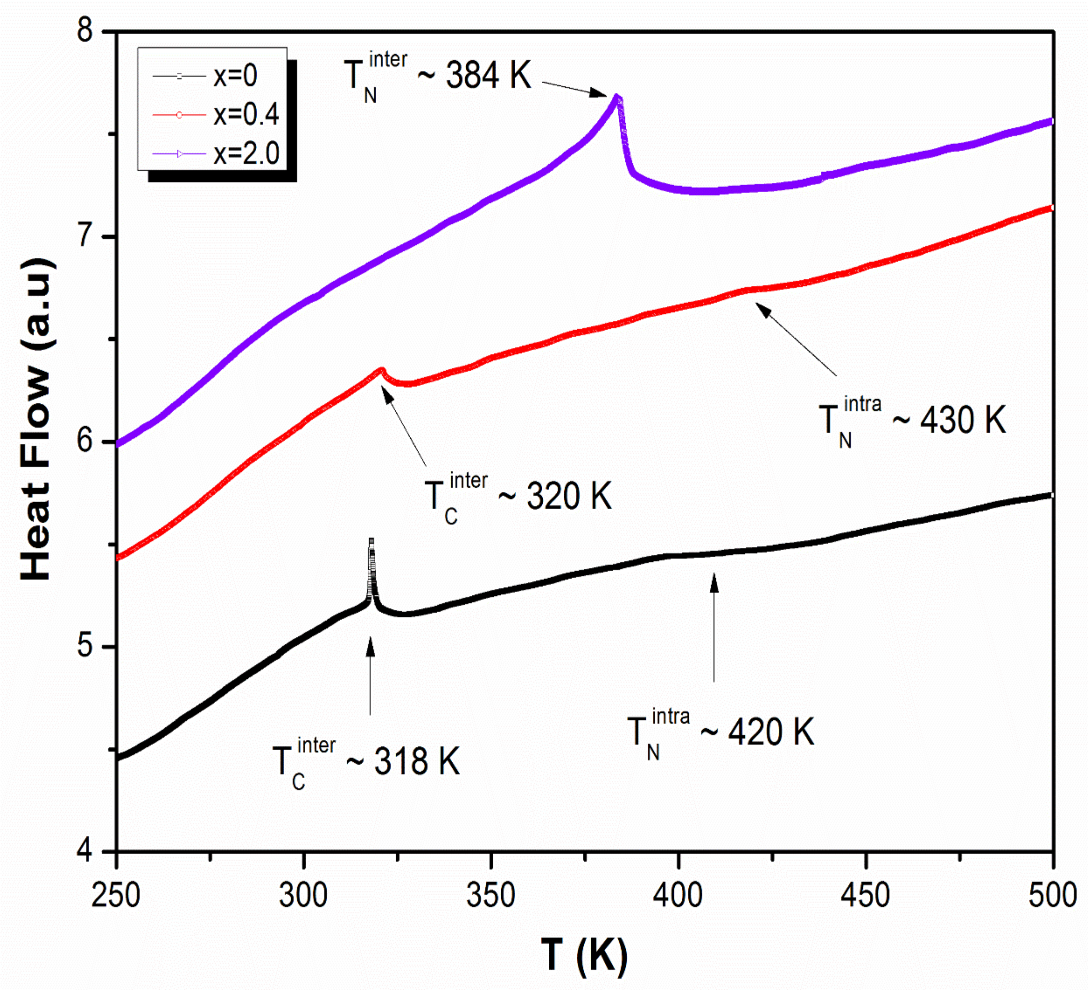

Figure S3 Differential scanning calorimetry measurements for CeMn2Ge2-xSix with x= 0, 0.4 and 2.0. The DSC data indicate the magnetic transition temperatures above room temperature respectively.

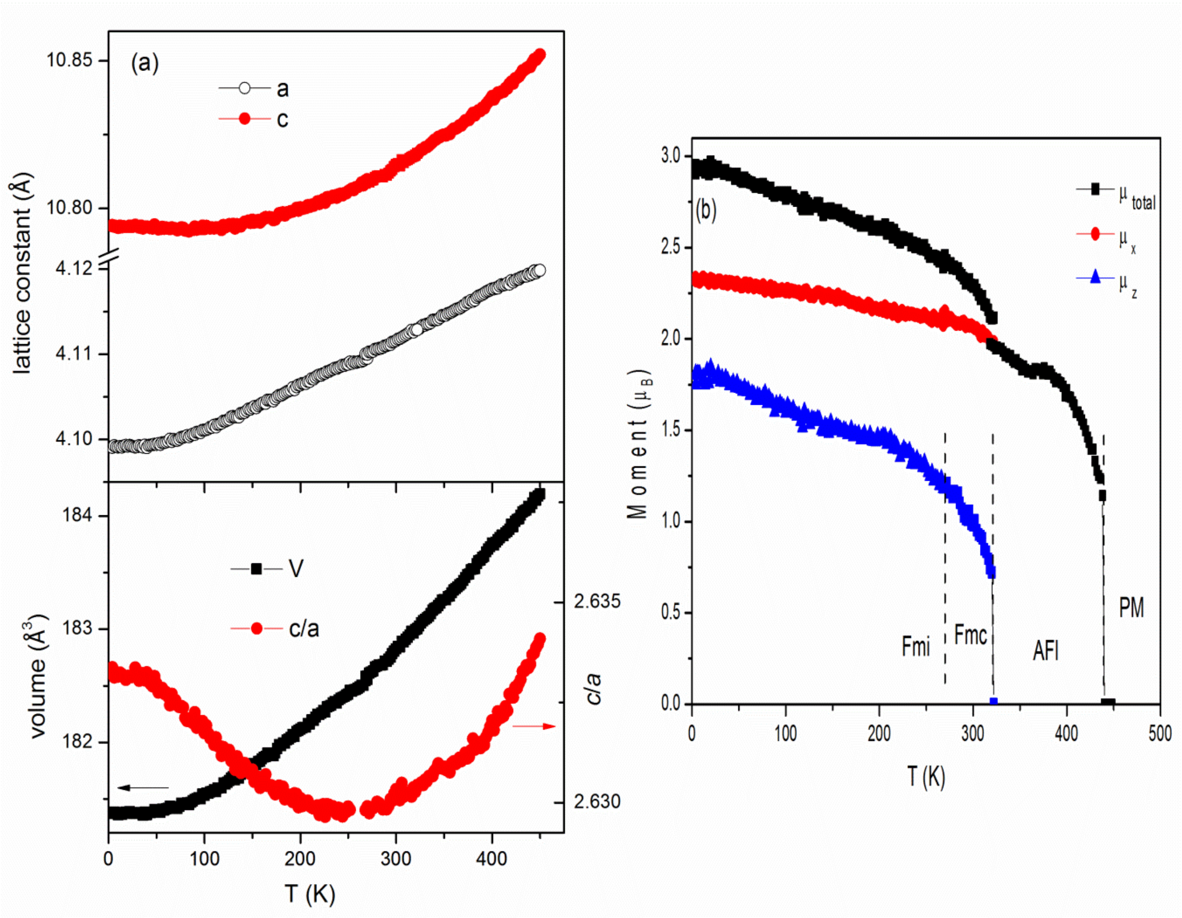

Figure S4 Structural and magnetic parameters for $CeMn_2Ge_{1.6}Si_{0.4}$ as derived from refinements of the neutron diffraction patterns: (a) Temperature dependences of lattice parameters *a, c,* unit cell volume and axial ratio *c/a*; (b) Temperature dependences of the magnetic moment and the propagation vector q_z (inset).

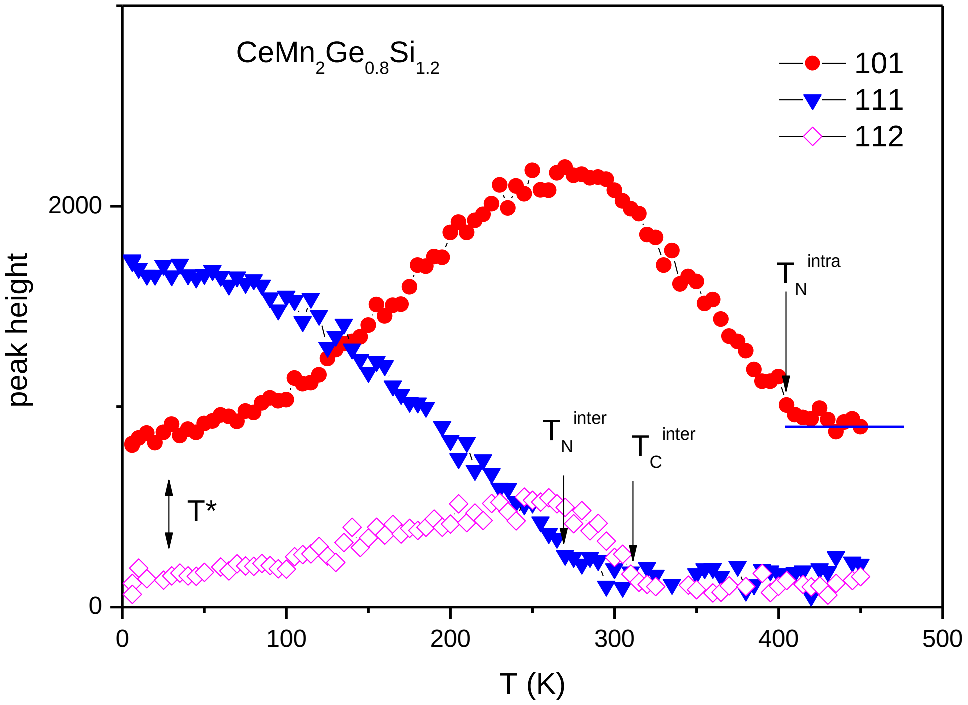

Figure S5 Temperature dependences of the intensities of the (101), (111) and (112) reflections over the temperature range 5-450 K for $CeMn_2Ge_{0.8}Si_{1.2}$. The various magnetic transition temperatures are indicated as discussed in the manuscript.

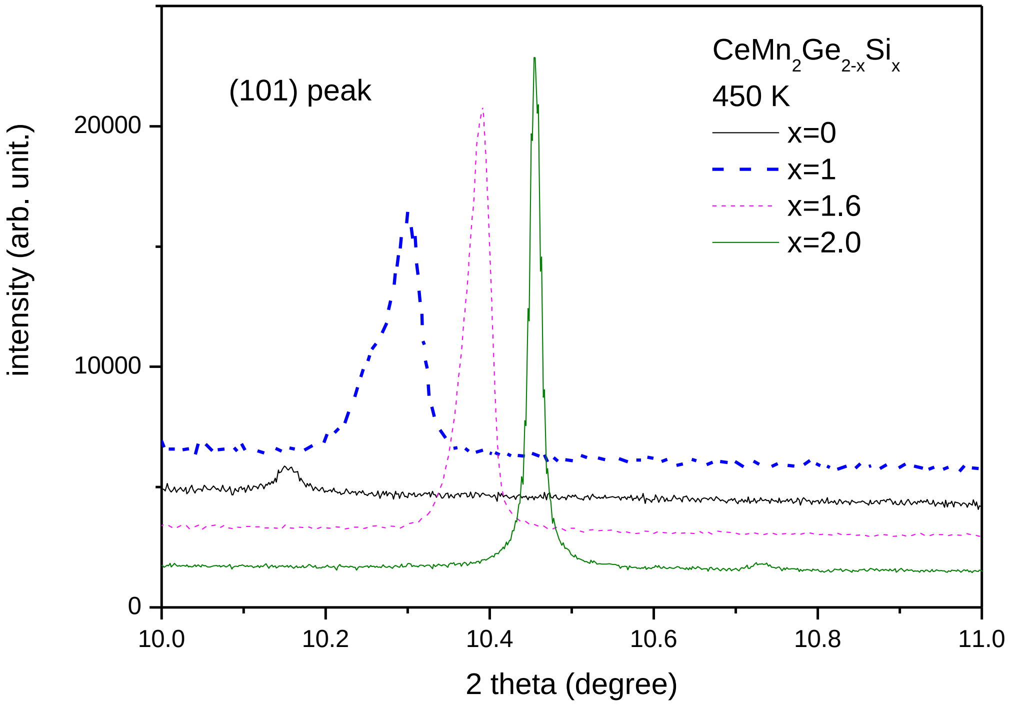

Figure S6 the reflections observed around the (101) peak position for $CeMn_2Ge_{2-x}Si_x$ compounds of Si concentrations x = 0.0, x = 1.0, x = 1.6 and x= 2.0 at 450 K (λ=0.6887 Å, Powder Diffract, Australian Synchrotron).

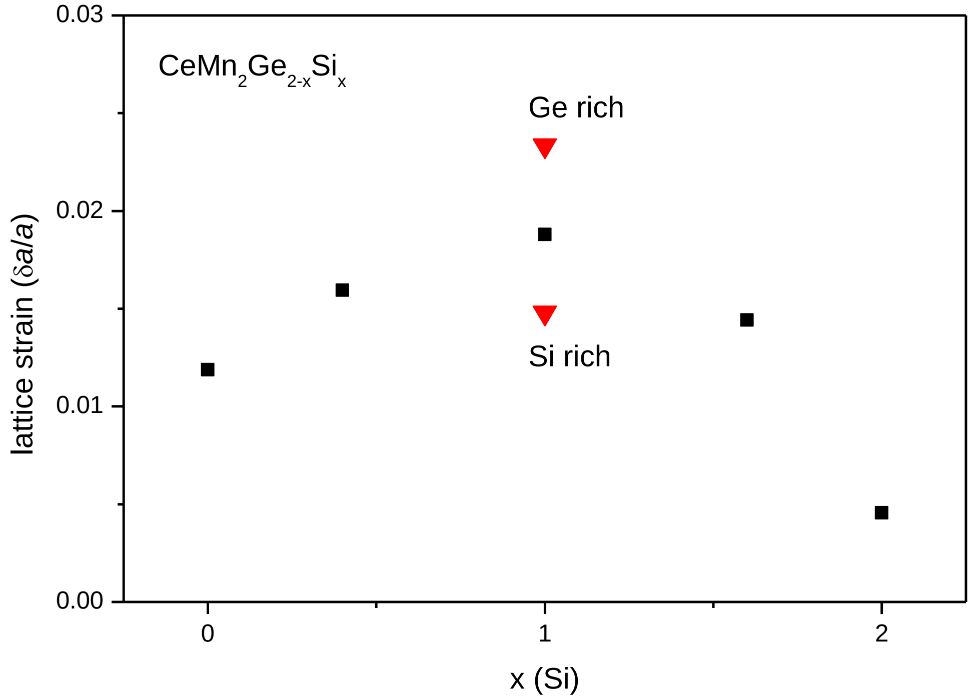

Figure S7 Lattice strain versus composition for $CeMn_2Ge_{2-x}Si_x$ compounds at 450 K. The values for the $CeMn_2Ge_1Si_1$ sample are shows as the average value (square symbol) as well as the Ge-rich and Si-rich regions (triangular symbol) as discussed in the text.
